# Borderline Personality Disorder Programme: Is the duration of the intervention related to clinical characteristics of patients?

**DOI:** 10.1192/j.eurpsy.2025.294

**Published:** 2025-08-26

**Authors:** J. T. Coelho, A. L. Cardoso, A. D. S. Moreira, C. S. Reis, H. Ginja, I. D. F. Pinto, I. M. Palma, E. Osório, R. Barranha

**Affiliations:** 1 São João Local Health Unit, Porto, Portugal

## Abstract

**Introduction:**

Borderline Personality Disorder (BPD) remains a challenging and complex disorder but it can be successfully treated.

In 2013, the first portuguese BPD specialized treatment programme was created in the Psychiatry Department at Local Health Unit of São João, becoming a very useful tool in the patients’ management.

**Objectives:**

We aim to study the influence of clinical characteristics on the duration of intervention and discharge from this BPD treatment programme.

**Methods:**

Retrospective observational study of patients admitted to this programme until August 31st 2024. Descriptive analysis of the results was performed using the SPSS software, version 29.

**Results:**

A total of 157 BPD patients were admitted to this programme, 154 (98%) of which are female. These patients had a mean age of 24,6 years old, when they engaged in the programme. During the follow‑up 39% have abandoned the programme, and 27% have been discharged due psychotherapeutic stabilization.

We will also present results related to association between clinical factors (namely previous diagnoses and history of use of substances) and the duration of the intervention/discharge plan, in order to know if our results are consistente with the ones from another centers where comorbidity with eating disorders and cocaine use disorder were clinical variables predictors of shorter duration of the intervention.

**Conclusions:**

Understanding the influence of clinical factors and their impact on intervention is essential to enhance this challenging programme to improve the treatment of BPD patients.

**Disclosure of Interest:**

None Declared

